# Activated Carbons from Thermoplastic Precursors and Their Energy Storage Applications

**DOI:** 10.3390/nano9060896

**Published:** 2019-06-19

**Authors:** Hye-Min Lee, Kwan-Woo Kim, Young-Kwon Park, Kay-Hyeok An, Soo-Jin Park, Byung-Joo Kim

**Affiliations:** 1 Research Center for Carbon Convergence Materials, Korea Institute of Carbon Convergence Technology, Jeonju 54852, Korea; hmlee2014@hanmail.net (H.-M.L.); 01090063344@hanmail.net (K.-W.K.); 2 Department of Chemistry, Inha University, Incheon 22212, Korea; 3 Department of Organic Materials & Fiber Engineering, Chonbuk National University, Jeonju 54896, Korea; 4 School of Environmental Engineering, University of Seoul, Seoul 02504, Korea; catalica@uos.ac.kr; 5 Department of Nano & Advanced Materials Engineering, Jeonju University, Jeonju 55069, Korea

**Keywords:** activated carbon, steam activation, low-density polyethylene, electric double layer capacitor

## Abstract

In this study, low-density polyethylene (LDPE)-derived activated carbons (PE-AC) were prepared as electrode materials for an electric double-layer capacitor (EDLC) by techniques of cross-linking, carbonization, and subsequent activation under various conditions. The surface morphologies and structural characteristics of the PE-AC were observed by field-emission scanning electron microscope, Cs-corrected field-emission transmission electron microscope, and X-ray diffraction analysis, respectively. The nitrogen adsorption isotherm-desorption characteristics were confirmed by Brunauer–Emmett–Teller, nonlocal density functional theory, and Barrett–Joyner–Halenda equations at 77 K. The results showed that the specific surface area and total pore volume of the activated samples increased with increasing the activation time. The specific surface area, the total pore volume, and mesopore volume of the PE-AC were found to be increased finally to 1600 m^2^/g, 0.86 cm^3^/g, and 0.3 cm^3^/g, respectively. The PE-AC also exhibited a high mesopore volume ratio of 35%. This mesopore-rich characteristic of the activated carbon from the LDPE is considered to be originated from the cross-linking density and crystallinity of precursor polymer. The high specific surface area and mesopore volume of the PE-AC led to their excellent performance as EDLC electrodes, including a specific capacitance of 112 F/g.

## 1. Introduction

Electric double-layer capacitors (EDLC) are reported to have better performance than secondary batteries in terms of power density (>4000 W/kg) and cycle stability (>100 k cycles), but have lower energy density (1 to 5 Wh/kg) [1,2,3]. As a result, many recent studies investigating EDLC have focused on increasing the energy density while maintaining their other advantages.

The critical factor for storing energy in EDLC is the physical electrostatic adsorption of electrolyte ions at the interface between the electrodes and the electrolyte [4,5]. Therefore, the ideal electrode materials for EDLC require a high specific surface area, optimal pore size distribution, and electrical stability for the fast transport of electrolyte ions, as well as high electrical conductivity for good electron movement [1,4,5]. Since the energy density of EDLC is proportional to the specific surface area of the active materials in the electrodes, many researchers have focused on developing activated carbon materials which can offer both high specific surface area and excellent electrical conductivity [1,5]. Nanocarbon materials such as carbon nanotubes and graphenes have high electrical conductivity, but they have the moderate specific surface area (under 300 m^2^/g) and are expensive and even low-density, resulting in low capacitance per unit volume [1,6]. Although activated carbons have specifically lower electrical conductivity than nanocarbons, they are the most widely used active materials for EDLC due to their excellent high specific surface area (compared to graphene [1,6] and carbon nanotube [1,7,8]), energy density, and price competitiveness [5,9].

Activated carbons (AC) can be produced in various ways, including the template method [10], a self-activation method [11,12], and physical/chemical activation methods [13,14,15,16,17,18,19,20,21,22,23,24]. The template method can easily control the pore structure (pore size and volume) of the resulting carbons, but it has a high process cost and low productivity. The self-activation method is a very simple process, but the specific surface area of the prepared AC is very low [11,12,13,14,15]. In commercial applications, AC is often produced by a physical activation method using crystallite oxidation and a chemical activation method using a dehydration reaction [7]. The chemical activation method can form more uniform pores and higher specific surface area with higher activation yields than the physical activation [14]. This is why many commercial AC are prepared using chemical activation methods [7]. However, despite the novel merits of chemical activation for the production of AC, a physical activation method may be more attractive for the mass production of AC for EDLC, because it offers a significantly lower price than the AC produced by chemical methods.

Commercial EDLC often uses organic electrolytes because of their high voltage characteristics and stability. Storing the ions of the organic electrolytes requires the AC electrodes to have pore diameters of 0.68 nm or greater [25,26,27]. Among the various physical activation methods, two kinds of activation agents—steam (H_2_O) and carbon dioxide (CO_2_)—are generally selected for this purpose. From our previous reports [28,29,30], the H_2_O activation method was preferred for producing electrode materials for EDLC because it can form relatively wide micropores compared to CO_2_ activation.

The activation process, which involves the carbonization and oxidation of precursor materials such as biomass [24], coals [21,22], polymers [16,17,18,19,20], and even fuels [7,13], is often affected by the crystallite structures of the precursors. Many researchers have suggested that if the crystallite structure of the precursor can be controlled, activated carbons with excellent pore characteristics can also be produced using a physical activation method, under proper process conditions [13,14,15,16,17].

Among various precursors, polymers have a specifically homogeneous crystalline structure, and the crystallite structures of carbonized polymers (intermediate materials for activated carbons) can be controlled during the stabilization and carbonization steps [29,31]. Studies on the use of conventional polymers as precursors for AC have typically utilized thermosetting polymers, which have highly aromatic ring structures [20]. However, the physical oxidation of large sized crystallites is quite difficult, due to the high thermal stability of graphite-like crystallite structures produced by the heavy aromatic ring structure during the carbonization process [16,17,29]. This is why most studies are carried out using the chemical activation process.

Kim et al. [31] used low-density polyethylene (LDPE) as a carbon precursor by cross-linking. LDPE has a long ethylene chain with a branched structure and high crystallinity compared to other precursors [16,19] The polymer chains in LDPE contain numerous branches of varying lengths that create substantial amounts of empty space between the polymer chains. In addition, LDPE is high-yield, low-cost, and contains very little ash.

This study investigated the synthesis of LDPE by the physical activation method to produce AC for EDLC with well-controlled pore characteristics by controlling the crystallite structure of the cross-linked and carbonized LDPE. Based on the results of previous studies [31], LDPE-based carbon precursors were prepared by a sulfuric acid cross-linking method. We prepared various low-density polyethylene derived activated carbons (PE-AC) and the electrochemical performance of the EDLC was measured in terms of the pore structure of the PE-AC.

## 2. Experiment

### 2.1. Materials and Cross-Linking

In this study, LDPE (LG Chemical Co, Seoul, Korea, LUTENE MB9205, 31,000–100,000 g/mol, 0.915 g/cm^3^) was used as a precursor and sulfuric acid (DaeJung Chem, 98%, 7683-4100, Gyeonggi-Do, Korea) was used as a cross-linking agent. The cross-linking of LDPE was performed in the same way as in a previous study [29]. LDPE was cross-linked under the sulfuric acid condition of 170 °C, which was the highest cross-link density in the previous study. The DSC peak (T_g_ or T_m_) of cross-linked LDPE disappeared by increased cross-linked density at 170 °C [31]. The cross-linked LDPE was washed with distilled water to pH 7 and then dried in an oven at 120 °C for 24 h. The cross-linked LDPE was set on an alumina boat which was then inserted in a self-made (SiC heater, length 1000 mm, diameter 100 mm) alumina tubular furnace. The cross-linked LDPE was heated up to the 900 °C under N_2_ flow at the rate of 10 °C/min and was held for 1 h at the carbonization temperature [31]. The carbonization yield of the carbonized LDPE was observed to be about 58.3%. Then, the gas flow was switched to H_2_O at a rate of 0.5 mL/min and held for 10 min (PE-H-9-1), 20 min (PE-H-9-2), 30 min (PE-H-9-3), or 40 min (PE-H-9-4). The PE-AC were cooled under nitrogen gas (300 mL/min).

### 2.2. Characterization

The morphologies and microstructures of the PE-AC were observed by field-emission scanning electron microscopy (FE-SEM, SU8220, Hitachi, Japan) and Cs-corrected field-emission transmission electron microscopy (Cs-corrected FE-TEM, JEM-ARM200F, JEOL, Tokyo, Japan), respectively. The X-ray diffraction patterns were collected using a X-ray powder diffractometer (X’Port PRO, PANalytical, Almelo, The Netherlands) with Cu Kα at a scan rate of 2°/min. Nitrogen adsorption isotherms of the PE-AC were measured with BELSORP-Max (BEL Japan, Tokyo, Japan) at liquid nitrogen temperature. All sample were degassed for approximately 6 h at 573 K, with the residual pressure maintained at 0.1 Pa, or less. The specific surface area was calculated using the Brunauer–Emmett–Teller (BET) method [32]. The micropore and mesopore size distributions were estimated via the nonlocal density functional theory (NLDFT) [33] and Barrett–Joyner–Halenda (BJH) [34] methods, respectively.

### 2.3. Electrochemical Tests

An electrode was made using the prepared PE-AC by mixing PE-AC:conductive material:binder = 8:1:1 wt.% using carbon black (Super-P, Timcal, Bodio, Switzerland) as the conductive material and binder of carboxy methyl cellulose (CMC, Dai-Ichi Kogyo Seiyaku Co., Ltd., Japan) & styrene-butadiene rubber (SBR, BM400B, Zeon, Japan) (ratio CMC:SBR = 2:1, m:m). For the preparation of EDLC electrodes, CMC was dissolved in water to obtain a 2.0 wt.% solution and equilibrated for 2 h at room temperature. The PE-AC and carbon black were added to the solution containing binder and dispersed with a planetary centrifugal mixer (AR-100, Thinky Co., Ltd., Tokyo, Japan) for 40 min. The so-obtained slurry was casted immediately on aluminium foil (20 μm, purity >99.9%) by using a laboratory scale doctor blade coater, whose blade was set at 125 μm. The coated foil was dried in an ambient atmosphere oven at 80 °C over night. EDLC were constructed using CR2032 coin cells. The samples were punched into round electrodes 12 mm in diameter. Two symmetric electrodes were isolated using a cellulose paper separator (NKK, Kanagawa, Japan). The electrolyte was 1 mol/dm^3^ tetraethylammonium tetrafluoroborate/propylene carbonate ((C_2_H_5_)_4_NBF_4_/C_4_H_6_O_3_). All electrochemical tests were performed at room temperature with a MACCOR Battery tester 4300 (Maccor Inc., Tulsa, OK, USA) and VSP electrochemical workstation (Bio-Logic Science Instruments, Grenoble, France). Galvanostatic charge/discharge tests (GCD) were performed at 2 mA constant current from 0.1 to 2.4 V. Cyclic voltammetry (CV) were performed in the same potential range of GCD at scan rates of 30 mV/s. The impedance plots were recorded in the frequency range 10 mHz to 300 kHz. The cells produced were measured based on the capacitance per unit weight, and calculated using only the weight of the active materials (F/g). The specific capacitance was calculated according to the GCD based on the following equation.

C_g_ = iΔt/mΔV (1)

where i is the discharge current (A), Δt is the discharge time (s), m is the mass of the electrode (g), and ΔV is the potential difference (V).

## 3. Results

### 3.1. Photo Images

**Figure 1:**
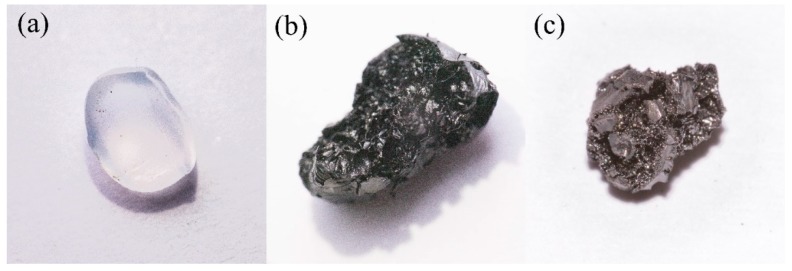
Photo image of (**a**) as-received low-density polyethylene (LDPE) pellet, (**b**) cross-linked LDPE sample, and (**c**) carbonized LDPE sample.

Figure 1 exhibits the surface changes in the LDPE pellets after cross-linking and carbonization, respectively. The LDPE pellet had a transparent color inherent to the polyolefin. The color of the cross-linked LDPE pellets changed to black and crater shapes were observed on the surface. For cross-linking, the LDPE pellets were exposed under sulfuric acid at 170 °C for 60 min. It is well-known that normal LDPE has a melting point of approximately 130–150 °C. Because the temperature of the sulfuric acid was higher than the melting point of the LDPE, the sulfuric acid could be easily inserted between the polymer chains (LDPE can be swelled at high temperature) and produce good cross-linking (condensation) [31]. During the cross-linking (condensation), domains which have low molecular weight (it is well-known that all polymers contain a broad range of molecular weights) can be oxidized under severe acidic conditions, resulting in the formation of the crater-like structures on the surface of the cross-linked LDPE samples. The surface of the carbonized LDPE samples (Figure 1c) was very similar to that of the cross-linked one. It was concluded that the polymer structure was well maintained even after carbonization because of good cross-linking.

### 3.2. FE-SEM Observation

H_2_O activation is a process of generating pores by causing the oxidation of the precursor’s crystallites by exposure to H_2_O vapor at a temperature higher than 900 °C. The surface morphology of the activated carbons is significantly changed by the oxidation of some crystallites on the surface. FE-SEM is a good analytical method for observing the morphology of activated carbon before and after the activation process.

**Figure 2:**
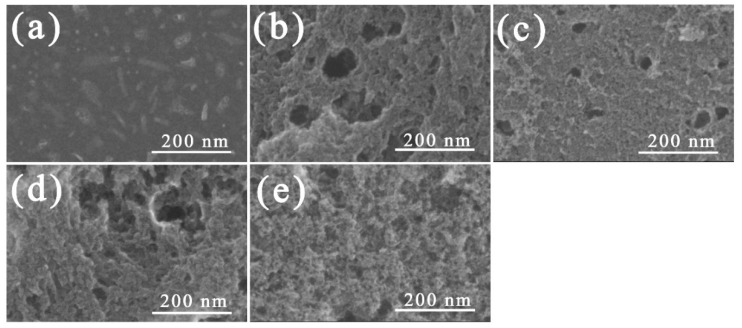
Field-emission scanning electron microscopy (FE-SEM) image of activated carbons with different activation times: (**a**) as-received (carbonized LDPE sample), (**b**) PE-H-9-1, (**c**) PE-H-9-2, (**d**) PE-H-9-3, and (**e**) PE-H-9-4.

Figure 2 shows the morphology of a carbonized LDPE sample and an PE-AC. Figure 2a is the SEM image of the carbonized LDPE sample. It is interesting to note that in Figure 1c many crater shapes can be observed in the carbonized LDPE sample, but the sample in Figure 2a has a smooth surface. This indicates that there was no pore development during the carbonization step. After H_2_O activation, the pore structures were observed in all samples (Figure 2b–e). As the activation time increased, the frequency of the pores (micro- and mesopores) increased gradually, and after 30 min of activation time many small pores were also found in inner areas, not only on surfaces.

This kind of pore development is typically not found in polymer-based AC because pore development normally proceeds from the surface to the inner area. It is possible that closed pores were formed at high temperature during the cross-linking or carbonization steps due to the swelled LDPE, and subsequently the closed pores and newly formed pores on the outer surface were finally connected with each other during the activation step.

In the sample exposed to 40 min of activation time, it was observed that the average size of the pores had been reduced and became homogenous. The explanation for this behavior is that the entire surface of the as-received sample was well-activated and the inner pores were fully exposed to the outer area at the same time. This is a clue that the specific surface area was dramatically enhanced after 40 min of activation time.

### 3.3. Cs-corrected FE-TEM Observation

**Figure 3:**
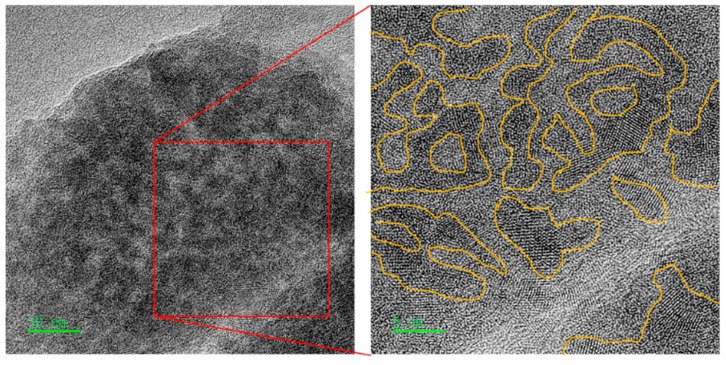
TEM image of carbonized LDPE sample.

Cs-corrected FE-TEM is a useful analytical method for observing the crystallite structure of carbonaceous materials. Figure 3 exhibits the crystallite structure of carbonized LDPE with amorphous domain and high graphitic domain complexes. In the magnified image (right), the dark part has a high graphitic domain and shows a layered structure similar to that of graphite, and it is confirmed that the bright part is amorphous domain or irregularly arranged planes (so-called hard carbon). Assuming that the bright part is oxidized to form pores, the expected pore shape can be very similar to the pore shape of the PE-AC observed in Figure 2.

We have reported in previous our studies [16,17] that amorphous or relatively small crystallines in the precursor structure are firstly oxidized in the preparation of AC through physical activation methods. This is the one of governing factors to determine average pore size and total pore volume of the resulting materials. Therefore, the LDPE precursor used in this study is presumed to have pores mainly due to oxidation of amorphous or relatively small crystallite. The crystallites portion (dark area) of the precursor may form pore walls in the manufactured AC. Thus, when observing the precursor on the Cs-corrected FE-TEM image in Figure 3, it can be assumed that the size of the developed pore is ~5 nm.

### 3.4. X-Ray Diffraction Analysis

**Figure 4:**
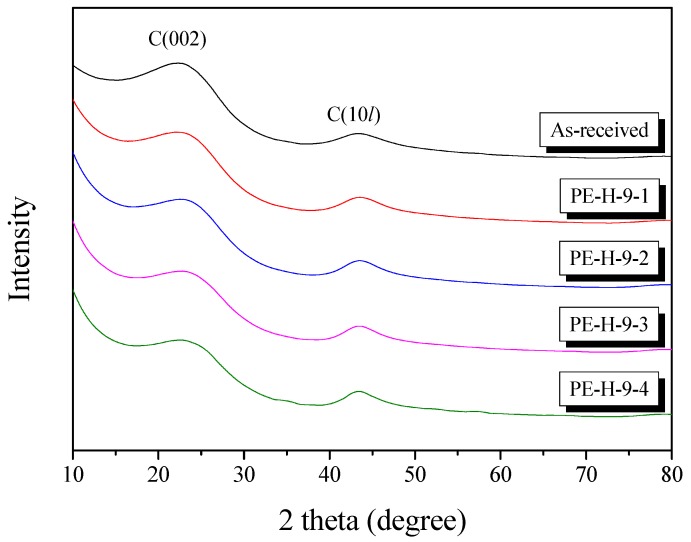
X-ray diffraction patterns of activated carbon for different activation times.

**Figure 5:**
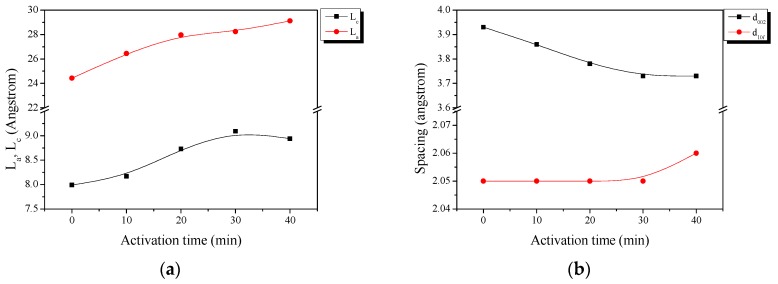
Structural characteristics of the activated carbons as a function of different activation times: (**a**) structural parameters and (**b**) interplanar distance.

X-ray diffraction (XRD) can easily analyze the crystallite structure of carbon materials. The crystallite structure of the AC was changed by oxidation of the precursor crystallite in the activation process. Figure 4 shows the results of the XRD analysis. As shown in Figure 4, all of the samples showed typical carbon peaks of C(002) and C(10*l*) at 2θ of 23° and 43°, respectively. Typically, graphite peaks are observed at 2 theta of 25°, but in this work C(002) were observed at 23°, indicating the carbon lattice was sparse and the lattice space was much wider than the highly crystallite materials. This type of XRD pattern is usually found in hard carbons or AC [14]. That is, AC is considered to have a mixed crystallite structure in which amorphous and graphitic domain are combined. Despite the increase in activation time, the C(002) and C(10*l*) peaks were well maintained. Table 1 and Figure 5 exhibited the fitting results of the XRD.

L_a_ (meaning crystallite size) was increased with increasing activation time, and L_c_ (meaning crystallite height) was increased up to 30 min of activation time. It is well known that amorphous regions and small graphitic crystalline in carbon precursors are preferentially oxidized [14,15]. Generally, the XRD data of carbon materials provides statistical data about the number of c crystallite aggregates rather than single crystalline. Therefore, the continuous increase in L_a_ can be considered to indicate a relative increase due to the oxidation of amorphous regions or small graphitic crystalline. It may also be that L_c_ was increased by the oxidization of amorphous regions or small layers of graphitic crystalline for up to 30 min, which were then maintained.

The d_002_ of the carbonized LDPE sample was 3.93 Å, which is higher than those of other polymers or carbon precursors; it is normally observed to be in the range of 3.54 to 3.69 Å [11,12,13,14,15,16,17,18]. The d_002_ may be relatively higher due to disordered lattice locations, caused by the sparse cross-linking density due to the aliphatic-based backbone of the LDPE precursors. As the activation time increased, the small crystallite lattices in the precursor were oxidized (removed) and then the total crystallite structure became denser, resulting in the decrease in d_002_. In PE-H-9-4 (the sample activated for 40 min), L_c_ was maintained and L_a_ was slightly increased. This means that large crystals were not oxidized further at the edge area (L_c_) and small crystallites were continuously removed during the activation process (L_a_).

### 3.5. N_2_/77K Adsorption Isotherm Analysis

**Figure 6:**
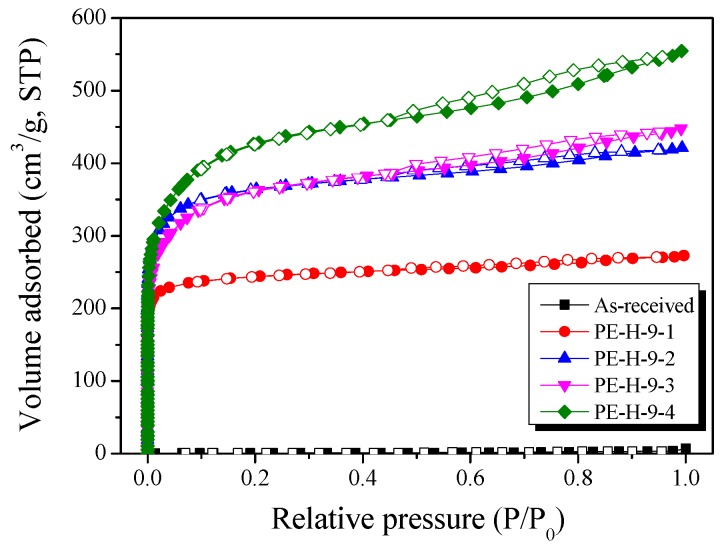
N_2_/77K adsorption isotherms of activated carbons as a function of different activation times.

In order to investigate pore development behaviors, N_2_/77K adsorption isotherms were employed and the results are exhibited in Figure 6. The textural properties of the PE-AC were also analyzed and are listed in Table 2. In Figure 6, every curve exhibited Type I according to the IUPAC classification [35]. This means that most of the pores are micropores. The activation yield tends to decrease with the increase in the specific surface area of the PE-AC, indicating the oxidation of tiny crystallite lattices, which led to the increase in the micropores of the PE-AC.

In PE-H-9-4, the specific surface area and total pore volume were observed to be 1600 m^2^/g and 0.86 cm^3^/g, respectively. It is interesting to note that the specific surface area predominantly increased up to 20 min of activation time and thereafter showed mild enhancement. Meanwhile, the mesopores gradually increased although the micropores were also increased. This result suggests the opening of the closed pores in the inner area which were self-formed during the cross-linking of the LDPE chains. In PE-H-9-3, the micropore volume decreased by 5% compared to PE-H-9-2, while the mesopores of PE-H-9-3 were significantly enhanced. This pattern can be attributed to the oxidation of some preformed micropores during further activation, which helped form the mesopores, resulting in the decrease in the specific surface area of the PE-H-9-3.

It was observed that the hysteresis of the isothermal adsorption curve became stronger when the activation time increased. Type D hysteresis was observed in all the samples, which means that the mesopores can be wedge shaped [36]. The increase in hysteresis is considered to be due to the fact that the internal pores of the precursor that formed during the cross-linking process were opened to the outside during the activation process.

On the other hand, the hysteresis of PE-H-9-3 was larger than that of PE-H-9-2, and the start of the knee was observed in the micropore ranges in the lower pressure zone. As described in Table 2, this suggests the micropores that formed earlier converted to mesopores during further activation.

**Figure 7:**
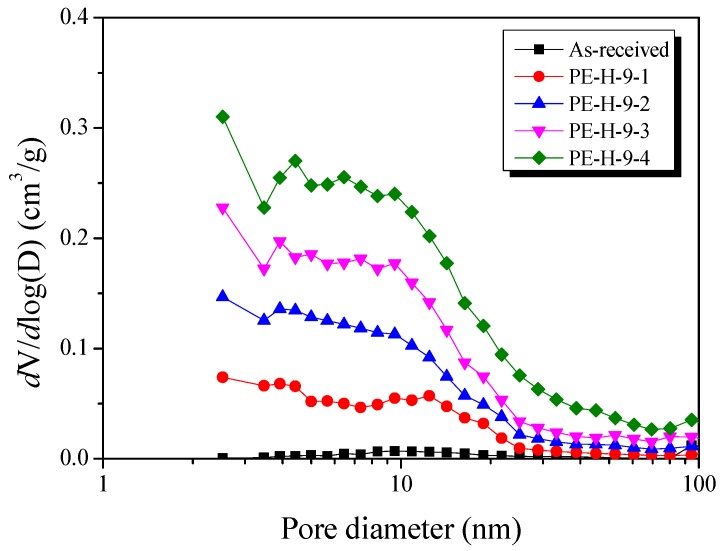
Pore size distribution of activated carbons with different activation times by Barrett–Joyner–Halenda (BJH) equation.

In order to observe the mesopores of the prepared PE-AC, the BJH equation was used to analyze the pore size distribution in the mesopore range. The results are shown in Figure 7. In general, the size distribution of the mesopores in PE-AC are observed to decrease gradually from 2 to 10 nm [13,14,15,16,17,18,19,20]. In the case of the PE-AC prepared in this study [21], it was observed that the mesopores were present over a wide region, up to 30 nm. In addition, as the activation time was increased, the volume of the mesopores proportionally increased. These results are consistent with the changes observed when activating precursors that have well-formed domain crystallite structures. These results indicate that the characteristics of the LDPE precursor are more easily attributed to mesopore formation, and that the amorphous or relatively small crystallites which were formed during the sulfuric acid cross-linking process for stabilization (not melted) are easily converted to the mesopore structure.

**Figure 8:**
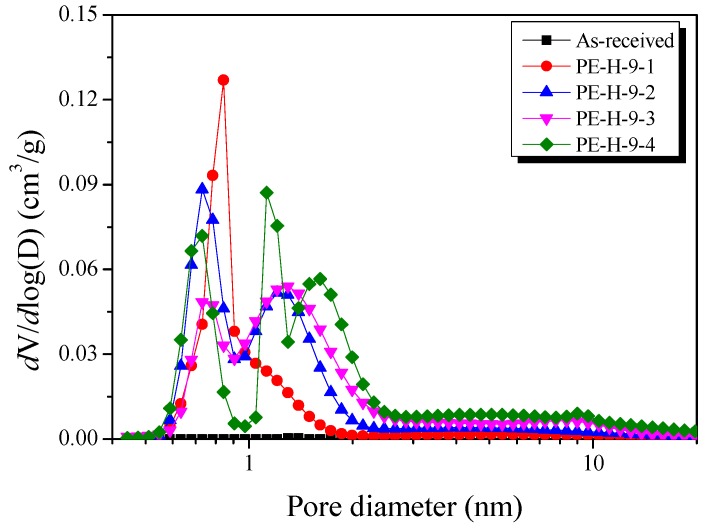
Pore size distribution of activated carbons with different activation times by nonlocal density functional theory (NLDFT) equation.

The NLDFT method was used to observe the conversion of the micropores to mesopores in Figure 8. In the initial 10 min activation (PE-H-9-1), it was observed that micropores with diameters of 1 nm or less predominantly developed. However, it was confirmed that the micropores decreased significantly in the 20 min-activated sample (PE-H-9-2), and the number of mesopores significantly increased.

It was observed that there were fewer micropores in PE-H-9-3 than in the PE-H-9-2 and the mesopores were increased. This result is consistent with Figure 6. For the most-activated sample (PE-H-9-4), it was observed that the mesopores were developed over a wider area.

From the above results, it can be concluded that (1) the development of micropores, (2) the conversion of mesopores to micropores, and (3) the development of additional micropores (or the external protrusion of internal pores) occurred complexly and simultaneously.

The above XRD and BET results suggest that the activation of the LDPE precursor has a unique characteristic. Generally, during activation using water vapor, the water vapor has difficulty penetrating into the precursor because water vapor has relatively large particles. As a result, the production yield and mesopore volume are not so high. However, in the present study, the activated sample (PE-H-9-4) had a production yield of 30%, despite having a mesopore ratio of 35% and a high specific surface area of 1600 m^2^/g. This is probably due to the fact that the internal pores generated inside the precursor during the cross-linking reaction of the LDPE were opened to the outside during the activation process, and this facilitated the penetration of the water vapor molecules. This reaction accelerated the oxidation of the small crystallites inside, and thus the L_c_ and L_a_ values were also increased.

### 3.6. Electrochemical Properties

**Figure 9:**
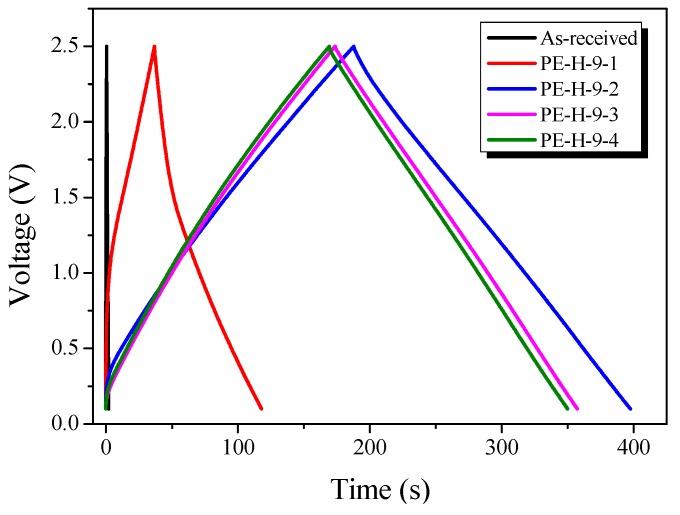
Charge–discharge curves of activated carbons with different activation times.

GCD measurements were performed to determine the capacitance of the activated LDPE fabricated electrodes. The linear voltage–time dependence demonstrates the typical capacitive behavior of the cell. Figure 9 exhibits the GCD curves of the EDLCs made from PE-AC. The as-received sample showed almost no GCD behavior, probably due to the lack of pore characteristics, as observed in Figure 6, Figure 7 and Figure 8. The GCD curves of PE-H-9-1 were observed to have few GCD times with nonlinear than the other PE-AC. These results indicate that the pore characteristics of PE-H-9-1 are inadequate for electrolyte ion storage and the capacitance is low.

The GCD behavior of PE-H-9-2 to PE-H-9-4 is highly reversible; the discharge curves display linear behavior and are approximately symmetrical to the corresponding charge curves. The linear behavior of the GCD curves demonstrates the excellent capacitive characteristics of the PE-AC. It is known that IR drop is dependent on the conductivity and pore structure of the electrode materials. IR drop does not appear in the GCD curves of the PE-H-9-2 to PE-H-9-4 because the samples had an optimal pore structure.

**Figure 10:**
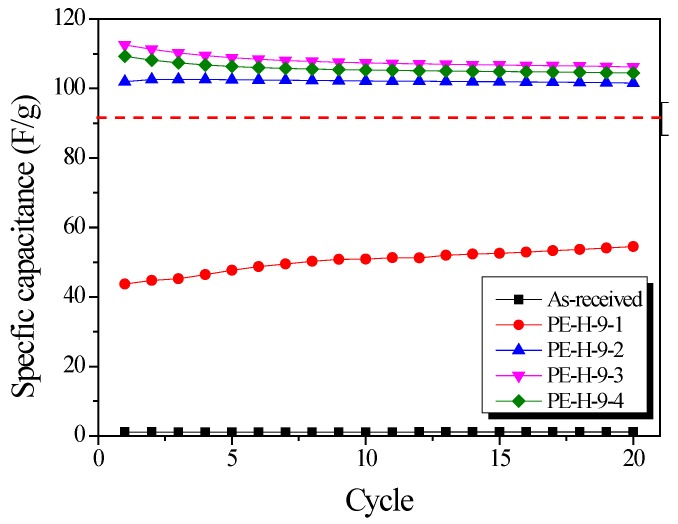
Variations in specific capacitance in relation to cycle number. The dotted red line denote the specific capacitance of commercial activated carbon for electric double-layer capacitor (EDLC).

Figure 10 shows the change in specific capacitance with GCD cycle. The specific capacitance increased with increasing activation time up to 30 min. This result is due to the increased pore volume and diameter of the pores in the PE-AC as activation time increases. The reduction in specific capacitance over 30 min seems to have reduced the pore volume available for ion storage space by increasing the pore diameter. Stable capacitance retention was observed in the electrodes after five cycles. The specific capacitance of the PE-AC was compared with YP-50F from the Kuraray company. The specific capacitances of PE-H-9-3 and PE-H-9-4 were over 110 F/g, higher than that of YP-50F [37].

**Figure 11:**
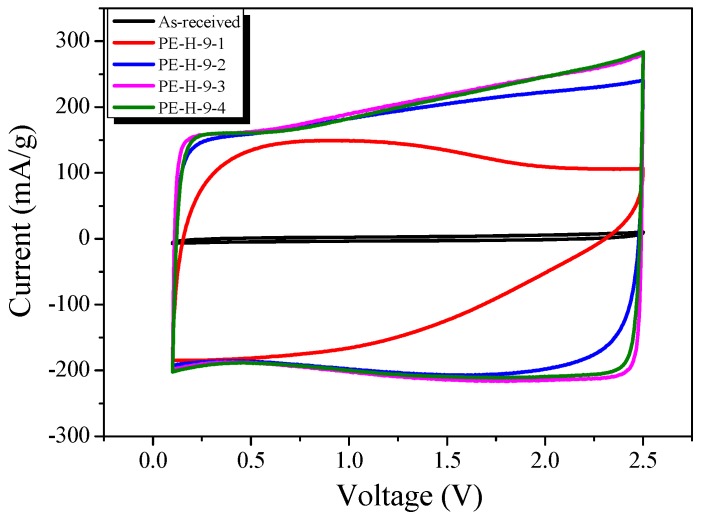
Cyclic voltammograms of activated carbons with different activation times.

The cyclic voltammogram (CV) curves show the charge–discharge behaviors of the PE-AC, and the EDLC has the ideal shape of a quadrangle. Figure 11 shows the CV for the EDLC made from the PE-AC. The CV curves in Figure 11 are very similar to the GCD curve results.

The EDLC made from as-received PE and PE-H-9-1 displays unfavorable electrochemical performance, with more distortion in the CV curve. These results can be attributed to the difficulty of ion migration in the pores of the PE-AC.

PE-H-9-2 to PE-H-9-4 exhibited a nearly rectangular shape from 0.1 to 2.5 V, which indicates efficient EDLC formation. This result implies rapid current response to the change in voltage from 0.1 to 2.5 V during charging and discharging. The specific capacitance values demonstrate an increasing tendency from as-received PE to PE-H-9-4. The current and area in the CV curve for PE-H-9-3 is apparently much larger than other PE-AC at the same scan rate. PE-H-9-3 shows the best specific capacitance compared to PE-H-9-2 and PE-H-9-4.

**Figure 12:**
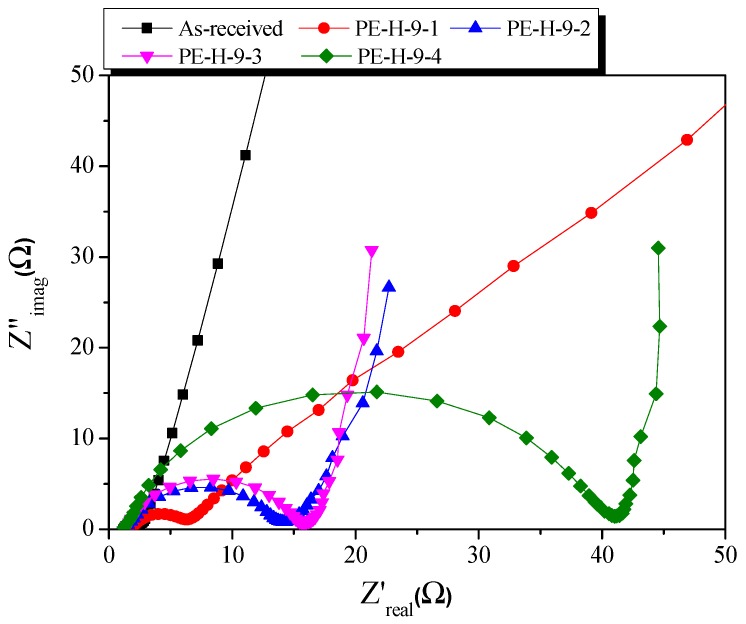
Nyquist plot of activated carbons with different activation times.

The Nyquist Plot is an analytical method that measures the equivalent series resistance (ESR) and Warburg resistance between electrodes and electrolytes. Figure 12 depicts the Nyquist plot of the EDLCs using PE-AC. In the Nyquist plot, the Warburg impedance of the EDLCs appears as a line with a 45° slope. The Warburg impedance of PE-H-9-1 was observed to be very long, because the movement of ions into the pores was not smooth. The impedance results for PE-H-9-1 showed the same trend as the charging and discharging results and CV curves. After 20 min of activation time, the impedance of the PE-AC were observed as the typical Nyquist Plot shape of EDLCs with consisting of semicircle and Warburg impedances. As the activation time increased, the size of the semicircle increased, but the Warburg impedance decreased. Warburg impedance is considered to be due to the smooth movement of ions due to the increase of pore diameter.

PE-H-9-4 was expected to have the best specific capacitance because the specific surface area and pore volume were observed to be the highest. However, PE-H-9-4 showed the largest semicircle in the Nyquist plot and was found to have high resistance. In conclusion, it is speculated that PE-H-9-4 has a similar specific capacitance to PE-H-9-2 and PE-H-9-3 due to its high resistance despite having the best pore properties.

The specific capacitance of activated carbons reported in the surveyed literature is presented in Table 3. Because different experimental methods were used by various authors to determine the specific capacitance, the values reported are sometimes not consistent. Table 3 shows the experimental results of our previous studies using the same experimental methods such as electrolyte (1 mol/dm^3^ ((C_2_H_5_)_4_NBF_4_/C_4_H_6_O_3_), CR2032 coin cell, voltage range, and scan rate. It was confirmed that the prepared PE-AC in this study had higher specific capacitance than other precursor-based activated carbon with the similar specific surface area. In addition, PE-AC has a specific capacitance similar to that of pitch-based activated carbon, whose specific surface area is nearly double the difference. These results suggest that PE-AC has a pore structure and low resistivity suitable for EDLC than other precursor-derived activated carbon.

## 4. Conclusions

The process of producing AC from LDPE by H_2_O activation was optimized and the textural properties and electrochemical characteristics of the produced materials were confirmed by applying them in EDLC. The structural properties of the LDPE and the activation conditions were found to significantly affect the performance of the electrode materials. A longer activation time led to an PE-AC with higher specific surface area and larger pore size, but it was also found that a too high pore diameter reduced specific capacitance.

The structural characteristics of the precursor LDPE led to the production of an AC with a mesopore-rich pore structure. Under optimal activation conditions, the resulting high specific surface area and mesopore-rich pore structure with suitable pore sizes resulted in PE-AC that are ideal candidates for EDLC electrodes. As the activation time increased, the specific surface area and mesopore volume of the PE-AC increased to 1600 m^2^/g and 0.30 cm^3^/g (35% of total pore volume), respectively. The PE-H-9-3 sample exhibited the highest specific surface area and the best electrochemical performance. These included the highest power, lowest ESR, and perfect reversible characteristics, as well as excellent maintenance of specific capacitance under high power operation. From the GCD characteristics, the PE-H-9-3 sample demonstrated the best results (112 F/g) of all the PE-AC.

## Figures and Tables

**Table 1:** Structural parameters of activated carbons with different activation times.

Sample	002 Peak				10*l* Peak			
	2θ	d_002_ (Å)	FWHM (2θ)	L_c_ (Å)	2θ	d_10*l*_ (Å)	FWHM (2θ)	L_a_ (Å)
As-received	22.61	3.93	10.15	7.99	44.10	2.05	7.18	24.43
PE-H-9-1	23.04	3.86	9.93	8.17	44.09	2.05	6.63	26.45
PE-H-9-2	23.52	3.78	9.30	8.73	44.09	2.05	6.27	27.97
PE-H-9-3	23.86	3.73	8.94	9.09	44.09	2.05	6.21	28.24
PE-H-9-4	23.84	3.73	9.09	8.94	44.00	2.06	6.02	29.12

**Table 2:** Textural properties of activated carbons with different activation times.

Sample	Activation Conditions	S_BET_^1^ (m^2^/g)	V_Total_^2^ (cm^3^/g)	V_Meso_^3^ (cm^3^/g)	V_Micro_^4^ (cm^3^/g)	Yield ^5^ (%)
As-received	-	3	0.01	0.01	0.00	-
PE-H-9-1	900 °C × 10 min	950	0.42	0.05	0.37	70
PE-H-9-2	900 °C × 20 min	1400	0.65	0.13	0.52	49
PE-H-9-3	900 °C × 30 min	1370	0.69	0.20	0.49	35
PE-H-9-4	900 °C × 40 min	1600	0.86	0.30	0.56	29

^1^ S_BET_: Specific surface area; BET method; $\frac{P}{v\left(P_{0}-P\right)}=\frac{1}{v_{m}c}+\frac{c-1}{v_{m}c}\cdot \frac{P}{P_{0}}$; ^2^ V_Total_: Total pore volume; BET method; ^3^ V_Meso_: Mesopore volume; BJH method: $r_{p}=r_{k}+t$ (r_p_ = actual radius of the pore; r_k_ = Kelvin radius of the pore; t = thickness of the adsorbed film); ^4^ V_Micro_: Micropore volume; V_Total_-V_Meso_; ^5^ Yield: $\frac{Weight\;of\;activated\;sample}{Weight\;of\;\mathrm{carbonized}\;\mathrm{sample}\;input}\times 100$

**Table 3:** Properties and characteristics of activated carbons as EDLC electrode materials with various precursor.

Precursor	Activation Condition	Specific Surface Area	Specific Capacitance	Electrolyte	Reference
LDPE	H_2_O, 900 °C, 30 min	1370 m^2^/g	112 F/g	1 mol/dm^3^ (C_2_H_5_)_4_NBF_4_/C_4_H_6_O_3_	In this work
Polyacrylonitrile	CO_2_, 1000 °C, 30 min	1530 m^2^/g	64 F/g		[30]
Polyacrylonitrile	KOH, 800 °C, 30 min	1160 m^2^/g	86 F/g		[20]
Coke	CO_2_, 1000 °C, 60 min	1040 m^2^/g	92 F/g		[21]
Pitch	H_2_O, 900 °C, 40 min	3230 m^2^/g	113 F/g		[13]
Polyurethane	CO_2_, 1000 °C, 60 min	1620 m^2^/g	105 F/g		[38]
